# Long-Term Effects of Maternal Deprivation on Cholinergic System in Rat Brain

**DOI:** 10.1155/2014/636574

**Published:** 2014-03-10

**Authors:** Branka Marković, Nevena V. Radonjić, Milan Aksić, Branislav Filipović, Nataša Petronijević

**Affiliations:** ^1^Faculty of Sport and Physical Education, University of Belgrade, Blagoja Parovića 156, 11000 Belgrade, Serbia; ^2^Institute of Clinical and Medical Biochemistry, Faculty of Medicine, University of Belgrade, Pasterova 2, 11000 Belgrade, Serbia; ^3^Institute of Anatomy “Niko Miljanić”, Faculty of Medicine, University of Belgrade, Dr. Subotića 4, 11000 Belgrade, Serbia

## Abstract

Numerous clinical studies have demonstrated an association between early stressful life events and adult life psychiatric disorders including schizophrenia. In rodents, early life exposure to stressors such as maternal deprivation (MD) produces numerous hormonal, neurochemical, and behavioral changes and is accepted as one of the animal models of schizophrenia. The stress induces acetylcholine (Ach) release in the forebrain and the alterations in cholinergic neurotransmitter system are reported in schizophrenia. The aim of this study was to examine long-term effects of maternal separation on acetylcholinesterase (AChE) activity in different brain structures and the density of cholinergic fibers in hippocampus and retrosplenial (RS) cortex. Wistar rats were separated from their mothers on the postnatal day (P) 9 for 24 h and sacrificed on P60. Control group of rats was bred under the same conditions, but without MD. Brain regions were collected for AChE activity measurements and morphometric analysis. Obtained results showed significant decrease of the AChE activity in cortex and increase in the hippocampus of MD rats. Density of cholinergic fibers was significantly increased in CA1 region of hippocampus and decreased in RS cortex. Our results indicate that MD causes long-term structure specific changes in the cholinergic system.

## 1. Introduction

Animal model of maternal deprivation (MD) is based on exposure to stress in early postnatal life. It has repeatedly been shown that early perinatal stress can cause various short- and long-term disturbances in cognitive, emotional, and other behavioral performances [1, 2]. Nonetheless, there is evidence that early stressful life events can increase the risk of developing schizophrenia [3–5]. Schizophrenia is a chronic, severe, and disabling brain disorder. Typical symptoms of schizophrenia can be divided into positive, negative, and cognitive ones. Typical antipsychotic drugs are effective in reducing the positive symptoms, but there is no efficacy against the negative symptoms and cognitive disorder [6–8]. Cholinergic system is a target for drug development aimed at improving treatments [9, 10]. Cholinergic disturbance in basal forebrain structures and their projections in schizophrenia could be notable for cognitive dysfunction given their known functional roles in conscious awareness and components of information processing, including attention, working memory, encoding memory consolidation, and retrieval [11, 12]. Recent studies show that selective muscarinic receptor agonist (xanomeline) can improve cognitive dysfunction in patients affected with schizophrenia [13].

The stress response includes acetylcholine (Ach) release in the forebrain, which plays an important role in many cognitive functions like learning [14, 15], attention [16], memory [17], and cortical modulation of sensory information [18]. This release of Ach is responsible for physiological and emotional responses, in particular through its action on the hypothalamic-pituitary system [19], one of the main physiological systems mediating the neuroendocrine response to stress [20]. Alterations in acetylcholine neurotransmission have been commonly reported in schizophrenia [21, 22].

The aim of this study was to examine long-term effects of maternal separation on cholinergic system by measuring AChE activity in different brain structures and density of cholinergic fibers in the hippocampus and retrosplenial (RS) cortex of rats.

## 2. Methods

### 2.1. Animals and Procedures

Male and nulliparous female Wistar rats at the age of 3 months were put together in standard Plexiglas cages with sawdust (26 × 42 × 15 cm), in a temperature controlled room (23 ± 1°C). The rats were on a standard 12 h light/dark cycle with lights on from 7:00 to 19:00 h, with water and food available* ad libitum*. Two weeks later, the males were removed and the dams were checked twice daily for delivery. The day of delivery was denoted as postnatal day (P) 0. On P 9, pups from four litters were subjected to the maternal deprivation procedure according to the previously published protocol [23]. Briefly, the mothers were removed from the cage around 10:00 a.m. and placed in a single cage housed in the same room as the pups. The pups were weighed and left in the home cage at room temperature. In the control four litters, mothers were removed at P 9, pups were weighed, and mothers were returned immediately (within 3 minutes). At P 10 (24 hours later), the deprived pups were weighed again and the mothers were placed back in the home cage. In the control litters, the mothers were again removed, the pups were weighed, and the mothers were placed back in the cage. All litters were later left undisturbed except for the routine cleaning of the cages, until P 21. On P 21, the litters were weaned. Rats were sacrificed at 2 months of age (P60). All experiments were performed on male animals.

All efforts were made to minimize animal suffering and reduce the number of animals used in the study. All experiments were carried out according to the NIH Guide for Care and Use of Laboratory Animals and were approved by the Local Bioethics Committee.

### 2.2. Brain Preparation for Measurements of Acetylcholinesterase Activity

Eight animals from control and eight from experimental groups were used for biochemical analysis of acetylcholinesterase activity. Four brain regions, dorsolateral frontal cortex, hippocampus, thalamus, and caudate nuclei, were dissected and the crude synaptosomal fraction was prepared according to the method of Whittaker and Barker [24–26]. Briefly, isolation of specific brain structures from individual animals was performed quickly on ice. Isolated tissue was homogenized in ice-cold buffer, pH 7.0, containing 0.25 M sucrose, 0.1 mM EDTA, and 50 mM K–Na phosphate buffer. Homogenates were centrifuged twice at 1000 ×g for 15 min at 4°C. The supernatant was further centrifuged at 20,000 ×g for 20 min. Supernatant obtained by this procedure represents crude synaptosomal fraction containing membrane vesicles (microsomes) from smooth and rough endoplasmic reticulum, Golgi and plasma membrane, and all of the soluble components of the cytoplasm.

### 2.3. Immunohistochemistry

For morphological analysis, five male animals from the control and five from experimental groups (P 60) were anaesthetized with chloral hydrate (3 mg/kg, i. p.) and transcardially perfused with fixative (4% formaldehyde in 0.1 M phosphate buffer solution). The brains were postfixed for 24 h at +4°C and cryoprotected by infiltration with sucrose for 2 days at 4°C (20% sucrose in 0.1 M phosphate buffer). Brains were frozen by immersion in 2-methyl-butane (Fluka) precooled to −80°C and stored at −80°C until cutting. Serial transverse sections (25-*μ*m-thick) were cut on a cryostat (Leica Instruments, Nußloch, Germany). Sections were collected on Super Frost Plus glass slides (Menzel, Braunschweig, Germany) in a standard sequence, so that four sections 250 *μ*m apart were present on each slide. Immunohistochemistry was performed according to a published protocol [27]. The commercially available goat anti-choline acetyltransferase antibodies (ChAT, 1 : 100; Chemicon, Hofheim, Germany) were used at optimal dilutions. Water-bath antigen unmasking was performed in 0.01 M sodium citrate solution, pH 9.0, for 30 min at 80°C [28]. Nonspecific binding was blocked using 5% normal serum from the species in which the secondary antibody was produced, diluted in phosphate-buffered saline, pH 7.3 (PBS), and supplemented with 0.2% Triton X-100 and 0.02% sodium azide for 1 h at RT. Incubation with the primary antibody, diluted in PBS containing 0.5% lambda-carrageenan (Sigma) and 0.02% sodium azide, was performed for three days at 4°C. After washing in PBS (three times for 15 min at RT), the appropriate Cy3 conjugated secondary antibody, diluted 1 : 200 in PBS-carrageenan solution, was applied for 2 h at RT. Following a subsequent wash in PBS, cell nuclei were stained for 10 min at RT with bisbenzimide solution (Hoechst dye 33258, 5 *μ*g/mL in PBS; Sigma). Finally, the sections were washed again, mounted in antifading medium (Fluoromount G; Southern Biotechnology Associates via Biozol, Eching, Germany), and stored in the dark at 4°C.

To estimate the densities of projecting immunolabeled fibers, pictures were taken on a fluorescent microscope (DM4000 Leica) with a 40x objective. Images from the RS and following subdivisions and layers of the hippocampus were taken: stratum moleculare, stratum granulosum, and stratum polymorphe of the dentate gyrus; stratum oriens, stratum pyramidale, and stratum lucidum of the CA3 region; and stratum oriens, stratum pyramidale, and stratum radiatum of the CA1 region. For each animal and layer, four pictures were taken. To estimate fiber density, the images were overlaid with a 1 cm stereological test grid (grid C4) using Photoshop 7.0 software (Adobe, San Jose, CA), and the number of intersections of fibers with the grid was counted.

## 3. Results 

### 3.1. The Acetylcholinesterase Activity Is Differentially Changed in the Cortex and Hippocampus of Maternally Deprived Rats

**Figure 1:**
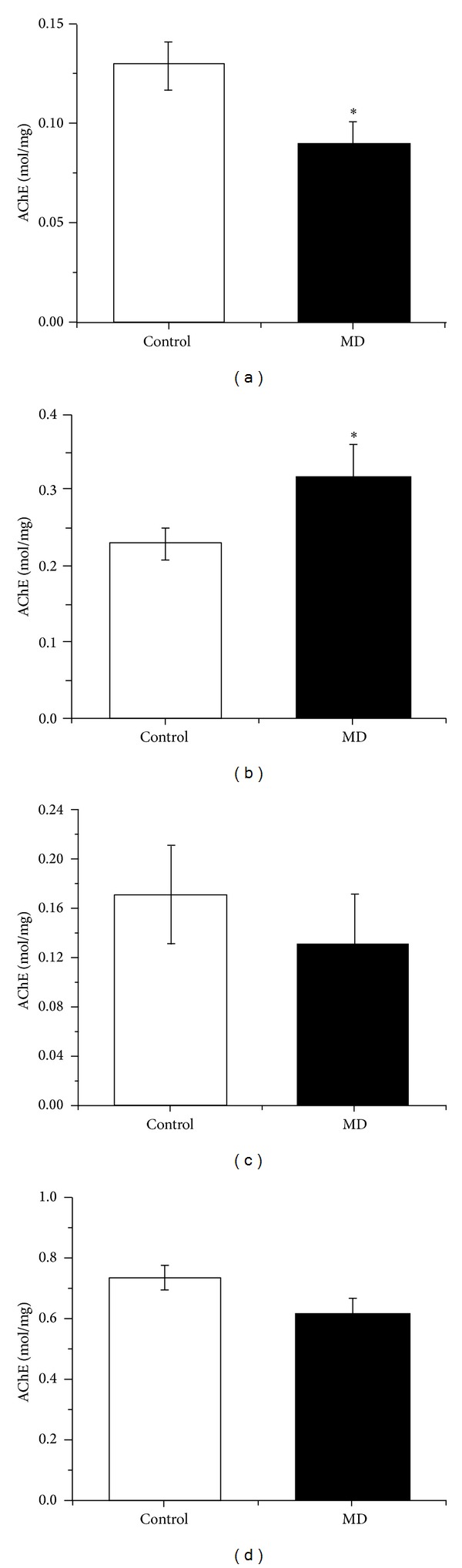
The activity of AChE in MD and control groups of animals (P 60) in synaptosomal fraction in cortex (a), hippocampus (b), thalamus (c), and caudate nuclei (d). Results are presented as mean ± SE. *P < 0.05.

The acetylcholinesterase activity in the synaptosomal fraction of cortex (Figure 1(a)) of MD rats was significantly decreased (*P* < 0.05) while in the hippocampus (Figure 1(b)) it significantly increased (*P* < 0.05) comparing to the values measured in the control group. In thalamus and caudate nuclei, no change in the acetylcholinesterase activity was observed (Figures 1(c) and 1(d)).

### 3.2. Immunohistochemistry Revealed Increase of the ChAT Positive Fibers Density in the Hippocampal CA1 Sector and Decrease in RS Cortex of MD Rats

**Figure 2:**
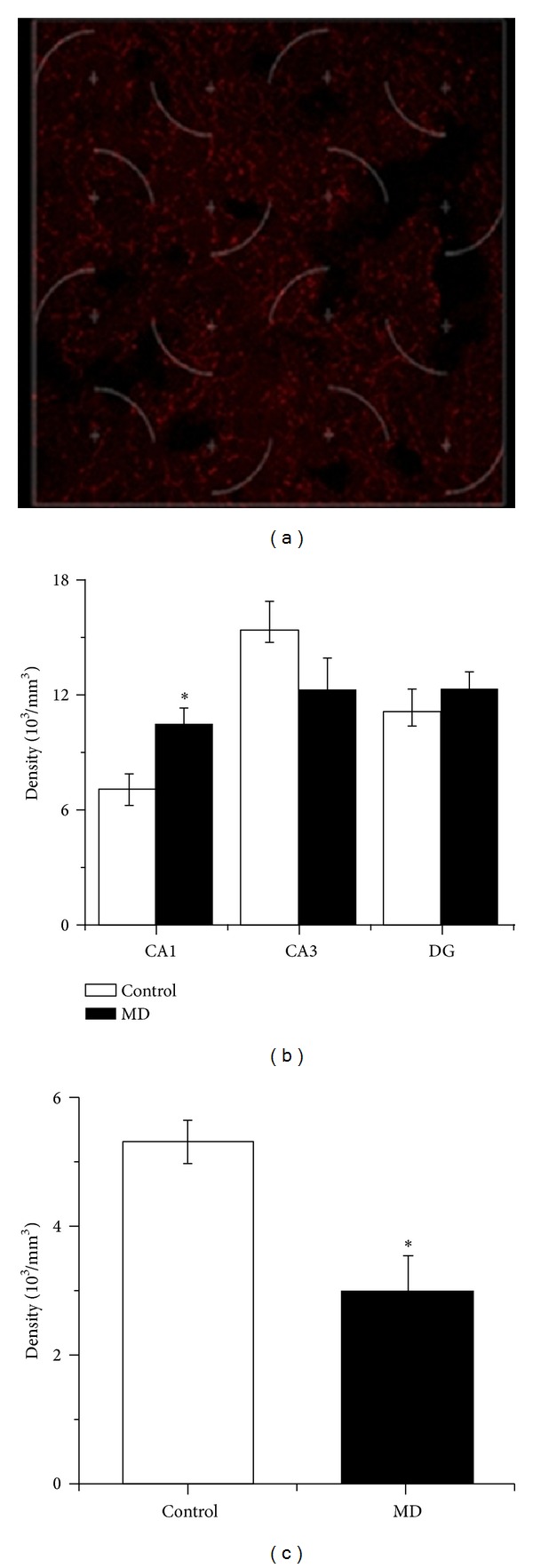
Representative immunohistochemical staining of the ChAT positive fibers in the hippocampus (a). Density of the ChAT positive fibers in the hippocampus (b) and RS cortex (c).

Representative immunohistochemical staining of the ChAT positive fibers in the hippocampus is presented in Figure 2(a). Measurements of ChAT positive fibers density have shown significant increase in CA1 region while no change in CA3 and DG was noticed (Figure 2(b)). The density of ChAT positive fibers in RS cortex was significantly decreased (Figure 2(c)) in MD animals.

## 4. Discussion

The results of our study have revealed region-specific changes in the AChE activity in cortex and hippocampus in the maternally separated rats. Also, the density of cholinergic fibers was decreased in the retrosplenial cortex but increased in CA1 region of hippocampus. These results are important since the hippocampus and cerebral cortex are critical areas to the processes of human's memory and cognition [29].

The cholinergic system has been proposed to contribute to the pathophysiology of schizophrenia probably as a result of an imbalance between central cholinergic and dopaminergic systems [30]. Growing body of evidence showed that patients affected with schizophrenia demonstrate cognitive deficit [31, 32] and cholinergic abnormalities, such as reduced acetylcholine in different brain structures [33] as well as widespread decrease in the level of muscarinic receptors in the postmortem brain [34–36]. Cholinergic involvement in schizophrenia is further supported by the fact that muscarinic antagonists can evoke a psychotic state (“antimuscarinic psychosis/syndrome”), which includes a range of cognitive and psychotic symptoms resembling schizophrenia [37]. Muscarinic cholinergic receptors (mAChRs) are G-protein-coupled receptors for the acetylcholine and consist of five different subtypes, termed M_1_–M_5_. Numerous preclinical and clinical studies with nonselective mAChR agonists suggest that activation of mAChRs improves cognitive function in patients suffering from various central nervous system disorders, and these studies, along with genetic studies, indicate that M_1_ is the mAChR subtype mediating the procognitive effects. Receptor protein and mRNA levels of M_1_ have been shown to be decreased in frontal cortex of schizophrenic patients. In addition, circulating antibodies against M_1_ have been found in the serum of schizophrenics, suggesting a link between the immune system and M_1_ in schizophrenics [38]. Nicotinic acetylcholine receptors (nAChRs) are ligand-gated ion channels, existing as combinations from a family of similar but distinct subunits *α*1–*α*10, *β*1–4, *γ*, *δ*, and *ε*. The most predominant receptor in the mammalian brain is the *α*4*β*2 nAChR, while there is also high expression of the *α*7 nAChR. Both receptors are widely expressed in areas of the brain important to cognition, such as the hippocampus, thalamus, frontal, cingulate, and occipital cortices. It has been proposed that the significantly higher levels of smoking seen in patients with schizophrenia might be due to an implicit desire to activate the *α*7 nAChR. Several lines of evidence suggest that the *α*7 nicotinic acetylcholine receptor (nAChR) could be an important pharmacological target for the treatment of cognitive deficits in schizophrenia. Polymorphisms in the promoter region of the *α*7 nAChR gene have been linked to sensory gating deficits in schizophrenia and some studies have found a reduced expression of *α*7 nAChRs in the frontal cortex of patients with schizophrenia [39].

On the other hand, disturbances of cholinergic system have been shown in animal models of schizophrenia. Treatment of adult rats with phencyclidine (PCP), antagonist of NMDA glutamate receptors, used to mimic some signs and symptoms of schizophrenia, was followed by increased efflux of acetylcholine [40] and alterations in the behaviors modulated by muscarinic receptors [41]. Furthermore, Du Bois et al. [42] have found changes in the expression of M1/4 receptors in the prefrontal cortex and hippocampus at different developmental time points following perinatal PCP treatment. Recently, Zugno et al. [43] have demonstrated that three hours of maternal deprivation daily, during first ten postnatal days (P 1 to P 10), caused an increase of AChE activity in prefrontal cortex, hippocampus, and striatum, as well as behavior alterations in adult animals. The authors have also investigated the effects of three different doses of ketamine in adult rats that were perinatally exposed to maternal deprivation and found that perinatal maternal deprivation made the animals susceptible to ketamine effects. In our study, the maternal deprivation on P 9, which lasted 24 hours, has produced different effects. We have noticed decrease of AChE activity in cortex and increase in hippocampus. Furthermore, our study has demonstrated significant increase of the ChAT positive fibers density in RS cortex and selectively in the hippocampal CA1 sector. The different pattern of AChE activity changes, seen in our study and the study of Zugno et al. [43], indicates that different protocols of maternal separations are reflected on the biochemical changes in the brain of adult animals. We can suppose that the different timing of early stress could be followed by different biochemical changes and interfere with developmental process. The alterations of AChE activity are particularly important since, independently of its catalytic function, AChE exhibits multiple biological actions on neuronal cell differentiation, adhesion, and neuritogenesis [44].

On the other hand, overexpression of AChE disrupts the glutamatergic system and results in damage of synaptic structures and excitatory function [45]. All of these findings indicate that excess of AChE that emerges under conditions of stress [46] could contribute to the pathophysiological processes developed after procedure of maternal separation.

Our study is in agreement with the findings of cholinergic abnormalities in schizophrenia and further supports the need for investigation of therapeutic strategies involving this neurotransmitter system. Whereas drugs, which have specific dopaminergic system as targets, are effective in reducing the positive symptoms in schizophrenia, they are not sufficient against the negative symptoms and cognitive disorder [47]. The neurotransmitter acetylcholine which plays an important role in the cognitive processes [11, 48] and learning and memory functions [49, 50] can play a key role in the development of new drugs. Use of acetylcholinesterase (AChE) inhibitors represents a promising possibility for treatment of cognitive disorder in schizophrenia and other psychiatric disorders [51].

However further experiments that will include the measurement of the density of cholinergic fibers in other cortical regions especially frontal cortex, the density of neuronal cell bodies in the nucleus basalis Meynert, and the density of mAChR and nAChR are needed for better understanding of the long-lasting cholinergic system changes initiated with early temporarily maternal separation.

## 5. Conclusion

Maternal deprivation causes region-specific changes of the AChE activity and cholinergic fiber density. In the cortex, the activity of AChE and the density of ChAT positive fibers were decreased while in the hippocampus AChE activity in whole structure and the density of ChAT positive fibers in CA1 region were increased. These results are important since the hippocampus and cerebral cortex are critical areas to the processes of human's memory and cognition and further support the need for investigations of therapeutic strategies involving cholinergic system in the treatment of schizophrenia.
